# Wait, treat and see: echocardiographic monitoring of brain-dead potential donors with stunned heart

**DOI:** 10.1186/1476-7120-10-25

**Published:** 2012-06-21

**Authors:** Marilena Casartelli, Tonino Bombardini, Davide Simion, Maria Grazia Gaspari, Francesco Procaccio

**Affiliations:** 1 Neuro Intensive Care Unit, University City Hospital, Verona, Italy; 2 Cardiology, University City Hospital, Verona, Italy; 3 CNR, Institute of Clinical Physiology, Pisa, Italy; 4 Institute of Clinical Physiology, National Research Council, Via Moruzzi,1, 56124, Pisa, Italy

**Keywords:** Brain death, Cardiac allograft acceptance, Echocardiography assessment, Heart transplantation, Hormonal treatment, Neurogenic stunning, Takotsubo cardiomyopathy

## Abstract

**Background:**

Heart transplantation is limited by a severe donor organ shortage. Potential donors with brain death (BD) and left ventricular dysfunction due to neurogenic stunning are currently excluded from donation – although such abnormalities can be reversible with aggressive treatment including Hormonal Treatment (HT) and deferred organ retrieval.

**Aim:**

To assess the recovery of left ventricular dysfunction in potential brain-dead donors with hemodynamic instability treated by aggressive treatment and HT.

**Methods:**

In a single-center, observational study design, we evaluated 15 consecutive brain-dead potential donors (DBD) (8 males, age = 48 ± 15 years) with hemodynamic instability. All underwent standard hemodynamic monitoring and transthoracic 2-dimensional echo (2-DE) with assessment of Ejection Fraction (EF). Measurements were obtained before BD and after BD within 6 h, at 24 h and within 48 h. HT (with insulin, methylprednisolone, vasopressin and T3) was started as soon as possible to treat hemodynamic instability and avoid administration of norepinephrine (NE). Eligible potential heart donors underwent coronary angiography.

**Results:**

After HT, we observed a normalization of hemodynamic conditions with improvement of mean arterial pressure (pre = 68 ± 8 mmHg vs post = 83 ± 13 mmHg, p < .01), cardiac index (pre = 2.4 ± 0.6 L/min/m^2^ vs post 3.7 ± 1.2 L/min/m^2^, p < .05), EF (pre = 48 ± 15 vs post = 59 ± 3%, p < .01) without administration of norepinephrine (NE) in 67% of cases. Five potential donors were excluded from donation (opposition, n = 3, tubercolosis n = 1, malignancy n = 1). At pre-harvesting angiography**,** coronary artery stenosis was present in 2 of the 10 consented donors. Eight hearts were uneventfully transplanted. No early graft failure occurred and all eight recipients were alive at 6-month follow-up.

**Conclusion:**

In BD donors, intensive treatment including HT is associated with improvement of regional and global LV function and reverse remodeling detectable by transthoracic 2DE. Donor hearts with recovered LV function may be eligible for uneventful heart transplant. The wait (in brain death), treat (with HT) and see (with 2D echo) strategy can help rescue organs suitable for heart donation.

## Introduction

Heart transplantation is an established procedure in end-stage heart failure patients, with satisfying long-term results. However, this surgical therapy is continuously limited by a severe donor organ shortage in recent years. Therefore, adequate and optimal utilization of all suitable donor organs is mandatory for increasing graft availability [1]. Evidence exists that certain ‘standard’ donor criteria can be significantly liberalized to increase the available donor pool by accepting ‘Marginal Donors’ who under conventional transplant guidelines would be declined as potential organ donors [2]. If echocardiography is the initial assessment investigation, the presence of segmental wall motion abnormalities with abnormal left ventricular ejection fraction in the absence of a history of heart disease is the single most common cause for exclusion criteria for donation [3], according to current eligibility criteria [4]. However, ventricular dysfunction may be transient [5], and arbitrary thresholds of LV function may exclude hearts that could eventually re-enter transplantable status. An early and aggressive Hormonal Treatment (HT), including triiodothyronine (T3), vasopressin, insulin and methylprednisolone has been proposed to manage unstable organ donors, particularly when cardiac function is impaired [1,6]. This study aimed to evaluate the effects of extended intensive treatment including HT and deferred harvesting in potential organ donors under conditions of hemodynamic instability.

## Methods

**Figure 1:**
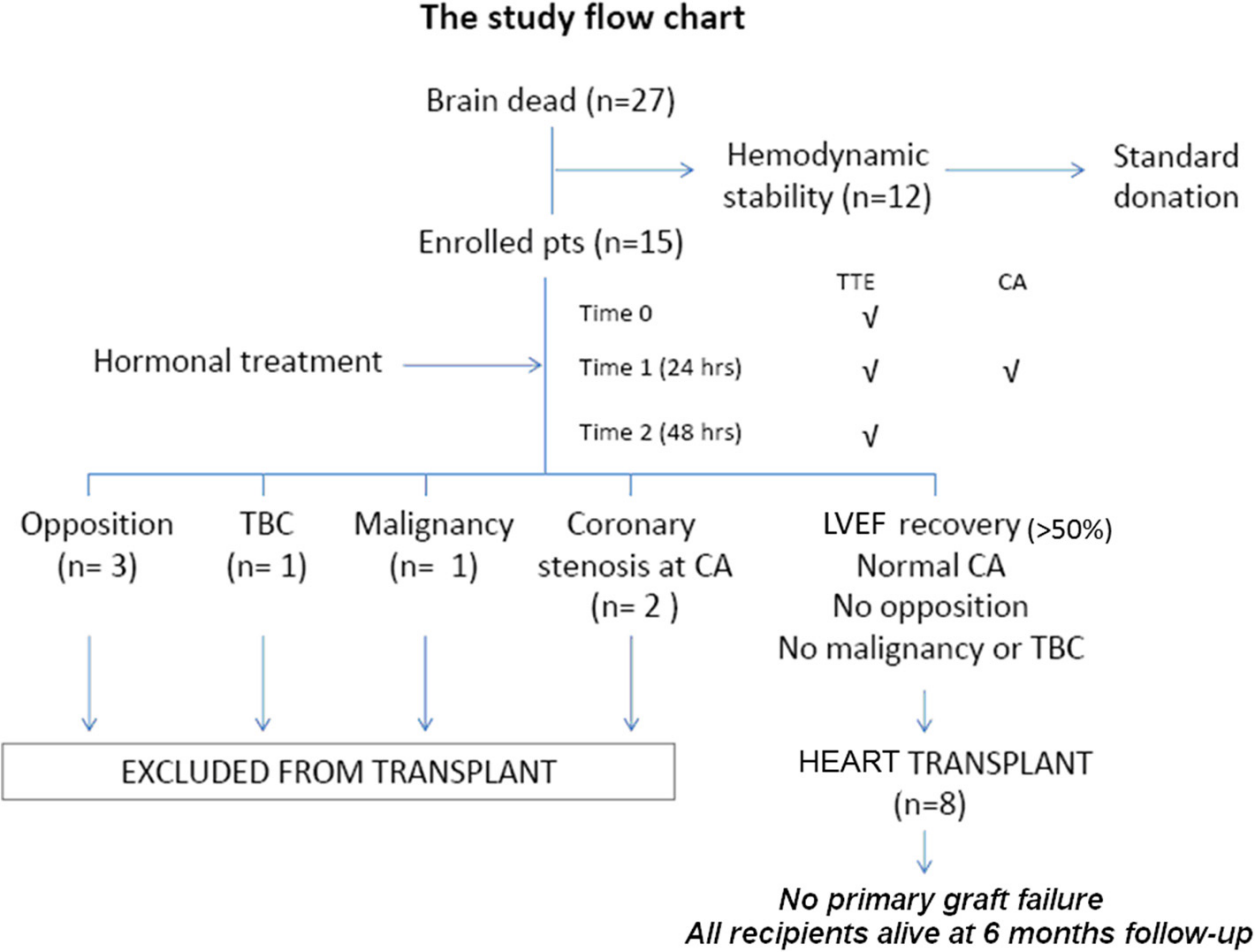
**The study flow chart.** TTE = transthoracic echocardiography, CA = coronary angiography.

From June 2010 through July 2011 we initially considered 27 consecutive brain-dead potential organ donors (DBD) with cerebral lesions of different etiology, mostly cerebral hemorrhage. Diagnosis of death was confirmed by strict adherence to standardized neurological criteria in accordance with Italian law and related guidelines. All donors were managed according to international guidelines [1,6]. From the initial population of 27 patients, 12 patients with hemodynamic stability underwent organ retrieval of eligible organs after brain death declaration and were excluded from the study. Thus, the final study population included 15 brain-dead patients, (8 men, 7 women; mean age 48 ± 15 years) under conditions of hemodynamic instability despite adequate fluid filling (Figure 1). The 15 potential donors were treated by HT and organ retrieval was delayed to 24–48 h from brain coning; retrieval was performed after legal declaration of brain death [7]. Arterial blood pressure and cardiac output were continuously monitored by the Vigileo/FloTrac system (software version 1.01; Edwards Lifesciences, Irvine, CA). Troponin values were recorded at brain death and later. The hormonal resuscitation was started: T3, 4 μg bolus + 1–3 μg /kg/h infusion; Vasopressin, 1 unit bolus + 0.5 – 4 U/h infusion; Methylprednisolone: 15 mg/kg bolus; Insulin, 1U/hr (titrate glucose 120–180 mg/dL) [8,9]. The hemodynamic management included: the use of intravenous fluids to maintain mean arterial pressure (MAP) > 60 mmHg, central venous pressure (CVP) 4–12 mmHg and urine output 1–2 ml/kg/h; correct anemia to maintain hemoglobin ≥ 10 g/dL; electrolyte replacement; ventilator management to achieve partial pressure of oxygen (PaO_2_) > 90 mm Hg; and pH in the 7.35 to 7.45 range, dopamine < 10 μg/kg/min. Norepinephrine was not administered, or infusion was weaned as soon as possible. All potential heart donors underwent an echocardiogram for evaluation of global ventricular function according to American Society of Echocardiography (ASE) recommendations [10]. The study complies with the Declaration of Helsinki, and the study protocol was submitted to the institutional ethics committee.

### Echocardiographic analysis

All potential donors underwent transthoracic echocardiography at baseline (within 6 h of brain death), and at the end of the first and the second day of hormonal treatment. We used commercially available equipment (Mylab ^TM^30 Gold Cardiovascular, Esaote, Italy) with a P4-2 MHz transducer. Two Neuro-ICU anesthesiologists trained in ultrasound performed the echocardiographic exams and eyeballing ejection fraction was assessed using apical 4- and 2-chamber views, adding a subcostal approach when useful to improve the examination quality [11]. The saved 2D cine loops were transferred for a later offline analysis using commercially available software. One experienced cardiologist unaware of the identity of the patient and blinded to all clinical data and previous readings estimated off-line the 2D LVEF by eyeballing method, before, during and after HT.

### Coronary angiography

Consented potential heart donors underwent coronary angiography. Multiple angiographic views were obtained for optimal visualization of the coronary arteries. For each identifiable lesion, the angiographer determined vessel diameter at the stenosis and at an adjacent angiographically normal reference site to quantify the percentage of stenosis diameter. Focal and non-circumferential atherosclerosis with 50% stenosis in proximal segments of at least one coronary vessel was regarded as coronary atherosclerosis.

### Transplant of eligible hearts

Eligible hearts (with normal echocardiography findings) were retrieved using standard technique and preserved with cold cardioplegic arrest and topical hypothermia (Figure 1). All transplants were performed using the bicaval anastomosis technique. Primary graft failure (PGF) after heart transplant was defined as need for immediate post-transplant mechanical circulatory support. The recipients followed routine treatment and follow-up procedures.

### Statistics

Software (SPSS 19 for Windows, SPSS, Chicago, Ill., USA) was used for statistical analysis. The statistical analyses included descriptive statistics (frequency and percentage of categorical variables and mean and standard deviation of continuous variables). Pearson’s χ2 with Fisher’s exact test for categorical variables and the Mann–Whitney test for continuous variables for intergroup comparisons were performed to confirm significance (using the Monte Carlo method for small sample comparisons).

## Results

Demographic, echocardiographic, laboratory and hormonal treatment data and outcome of individual potential donors with hemodynamic instability are shown in Table 1. The cause of death was cerebral hemorrhage in nine cases, ischemic stroke in one case and head trauma in five cases. Median maintenance time was 48 h (30–156 h). Early increase in troponin was observed in 93% (mean 3.36 ± 4.1 ng/mL) vs a final value of 0.48 ± 0.6 (p < 0.05 vs baseline). After HT, we observed a normalization of hemodynamic conditions with a clear improvement of systolic blood pressure (pre = 95 ± 10 mmHg vs post = 124 ± 15 mmHg, p < .01), mean arterial pressure (pre = 68 ± 8 mmHg vs post = 83 ± 13 mmHg, p < .01), cardiac index (pre = 2.4 ± 0.6 L/min/m^2^ vs post 3.7 ± 1.2 L/min/m^2^, p < .05), peripheral arterial blood lactate (pre = 3.2 ±1.8 vs post = 1.6 ± 0.7 mmol/L, p < .01). In 10/15 cases norepinephrine was never used after brain death; in 5/15 cases norepinephrine infusion was stopped within 12 h. Transthoracic echocardiography was feasible and interpretable in all brain-dead potential donors.

**Table 1:** Potential donors with hemodynamic instability

	**Sex**	**Age**	**M** hours	**Early LVEF** %	**Final LVEF** %	**Peak Troponin***ng/mL*	**Final Troponin***ng/mL*	**T3** hours	**NE** hours	**Outcome**	**Heart Donor**	**Other Organs**
1	M	32	**51**	**60**	**60**	0.47	0.26	15	-	Donor	**Yes**	2 L-2 K-Li
2	F	54	**30**	**60**	**60**	3.27	0.50	15	-	Donor	**Yes**	2 K
3	M	66	**56**	**60**	**60**	2.82	0.87	15	12	Donor	CAD	2 K-Li
4	M	51	**30**	**55**	**55**	0.99	0.64	12	-	Donor	CAD	2 K-Li
5	M	20	**36**	**60**	**67**	2.70	0.30	33	-	Opposition	-	-
6	M	48	**39**	**55**	**60**	0.05	0.015	27	6	Donor	**Yes**	2 L-2 K-Li-P
7	F	51	**33**	**60**	**60**	11.40	2.15	24	-	Donor	**Yes**	2 K-Li
8	F	56	**72**	**60**	**60**	5.61	0.25	63	-	Donor	**Yes**	2 K
9	F	45	**48**	**37**	**55**	0.23	0.21	9	-	Donor	**Yes**	2 K-Li
10	M	66	**90**	**40**	**55**	4.35	0.05	3	-	Opposition	-	-
11	M	16	**114**	**30**	**60**	0.90	0.08	18	12	Opposition	-	-
12	F	64	**69**	**40**	**60**	2.17	-	27	12	Donor	**Yes**	2 L-2 K-Li
13	F	56	**48**	**20**	**54**	13.80	0.90	12	-	Tubercolosis	-	-
14	F	46	**156**	**23**	**60**	0.38	0.17	60	3	Donor	**Yes**	2 K
15	M	50	**42**	**55**	**58**	1.32	0.30	3	-	Malignancy	**-**	**-**

M = maintenance; CAD = Coronary Artery Disease; LVEF = Left Ventricular Ejection Fraction; T3 = Triiodothyronine; NE = Norepinephrine; Li = Liver; L = Lung; K = Kidney; P = Pancreas.

### Echocardiographic data

**Figure 2:**
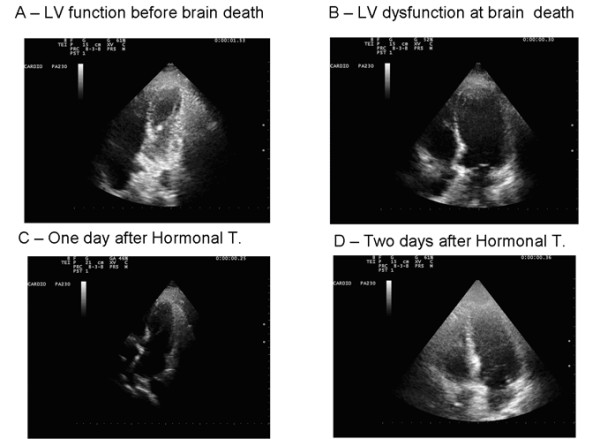
**Serial four chamber cineloops of a potential heart donor with hemodynamic instability.Left upper panel** Normal LV function before brain death. **Right upper panel**. Takotsubo-like LV dysfunction immediately after subarachnoid hemorrhage and brain death: echocardiographically detected left ventricular systolic dysfunction excludes this heart from transplant according to standard criteria.** Left lower panel** Reduction of end-systolic volume and improved systolic thickening. One day after early and aggressive hormonal treatment, including triiodothyronine, vasopressin, insulin and methylprednisolone with improvement of heart function. **Right lower panel**. Two days after hormonal treatment the heart definitively normalized, was retrieved and was successfully transplanted. An additional movie file shows this in more detail [see Additional file 1].

**Figure 3:**
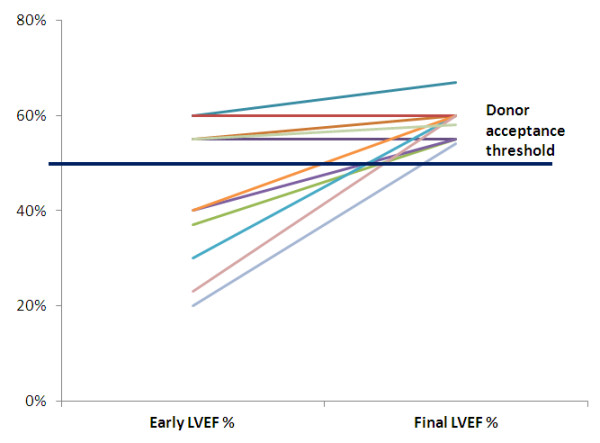
**The values of left ventricular ejection fraction at study entry (Early LVEF %), and following 2 days (Final LVEF %) of hormonal treatment.** At individual potential donor analysis, the improvement was more obvious in hearts with worse function at baseline.

A typical pattern of recovery is shown in Figure 2. The patient showed severe lef t ventricular dysfunction and dilation at study entry and full recovery after HT. We observed a progressive improvement of indices of global function (LVEF pre = 48 ± 15 % vs post = 59 ± 3%, p < .01). At individual patient analysis, the improvement was more obvious in six patients with worse function at baseline (Figure 3). At segmental analysis, wall motion abnormalities at study entry more often involved the apical and septal (n = 2 ) walls, the global left ventricle (n = 3), and the inferior wall in one case.

### Coronary angiography data

At pre-harvesting angiography**,** coronary artery stenosis was present in two of the ten consented donors, both with normal EF at study entry (Table 1). All consented donors with abnormal LV function at study entry showed normal coronary arteries at angiography. In one consented donor coronary angiography was not performed because of the donor’s young age (32 years old).

### Transplant eligibility

At the end of treatment all patients showed normal regional and global LV function and were eligible for heart donation. In patients with normal function at study entry, five out of nine potential heart donors became effective donors. The remaining four were excluded – due to opposition in one, to coronary stenosis at angiography in two and to malignancy in one. In patients with abnormal function at study entry, three out of six potential heart donors became effective heart donors. The remaining three were excluded for opposition in two, tuberculosis in one. A total of eight consented hearts without co-morbidities were uneventfully transplanted. No early graft failure occurred and all eight recipients were alive at 6-month follow-up.

## Discussion

In Italy about 2300 patients per year are declared brain dead, out of around 5500 dying in ICUs from acute devastating cerebral lesions (i.e., brain trauma, hemorrhagic or ischemic stroke and anoxia). Currently, brain-dead consented organ donors number around 1100 with less than 300 hearts transplanted per year (data from the “Italian National Health Institute ”, 2011). This severe shortage in transplantable heart status depends on eligibility criteria based on the donor age, which is continuously increasing, and functionality, which may be acutely affected by the consequences of brain coning and hemodynamic instability. Thus, new strategies enhancing functional recovery and reliable prediction of successful heart transplantation are needed. In brain-dead potential donors with hemodynamic instability, echocardiographic serial assessment of LV function is feasible and shows, in 40 % of patients, a moderate-to-severe alteration in global and regional LV function. These alterations may be fully reversible upon intensive care, in a couple of days. In this setting, regional wall motion abnormalities can occur in the absence of underlying coronary artery stenoses, and also coronary artery stenoses can be present with normal regional wall motion [12,13]. In this single-center study, based on a limited number of potential donors, we showed that serial echocardiography is feasible and interpretable in the extreme setting of DBD potential donors, and at least as a proof-of-concept it can be helpful, together with coronary angiography, in selecting suitable heart donors otherwise dismissed on the basis of the initial assessment. When initial LVEF is impaired, the period of treatment after brain death can be intentionally prolonged towards the golden time for harvesting, facilitating recovery of stunned hearts and eventually improving the quality of the graft before transplantation. Early hormonal treatment may facilitate complete weaning from vasopressors, and the recovery of stunned hearts often occurs within the maintenance period. These potential donors can manifest ECG abnormalities, hemodynamic instability, increased troponin and left ventricular segmental wall motion abnormalities mainly due to adrenergic storm and not to intrinsic cardiac disease. However, according to current eligibility criteria [4], the presence of segmental wall motion abnormalities of the left ventricle is an exclusion criterion for donation. In a large subset of brain-dead donor hearts left ventricular performance is reduced because the myocardium is regionally stunned or hibernating rather than irreversibly infarcted or fibrotic [12,13]. The detection of reversible dysfunctional myocardium is clinically relevant, as regional or global left ventricular function will improve after transplant [14,15]. Such recovery can be facilitated, elicited by cardiovascular targeted intensive treatment, including hormonal treatment and noradrenaline sparing strategy, lasting until 24–48 h before harvesting [8-10,16,17]. Thus, the challenge is to identify markers that indicate which hearts are likely to have good function or be treated to satisfactory hemodynamic status and transplantation suitability.

### Serial echocardiography in the stunned brain-dead potential donor

Standard rest echocardiography is a non-invasive, portable, and rapidly available investigation that is ideally suited to the accurate assessment of donor ventricular function. However, in the presence of left ventricular dysfunction, a “single spot” echocardiography does not predict reversibility or non-reversibility of ventricular function after heart transplant [14]. If echocardiography is the initial assessment investigation, echocardiographically detected left ventricular systolic dysfunction in the absence of a history of heart disease is the single most common cause for non-transplantation of an organ [15]. However, ventricular dysfunction may be transient [14], occurs in 10% to 42% of donor hearts, and arbitrary thresholds of LV function may exclude hearts that could be resuscitated to transplantable status. Such limitations can be overcome with serial evaluations allowing us to identify those hearts with reversible dysfunction that may recover transplantation status.

### Hormonal treatment in the stunned brain-dead potential donor

The brainstem ischemia occurring in the terminal phase of the pathophysiological process which leads to brain death may cause an “autonomic storm” with intensive sympathetic nervous system activity, followed by vasoparesis and hypotension [13]. Such injury might be exacerbated by changes in endocrine homeostasis, metabolism, and the development of a proinflammatory state. In patients with subarachnoid hemorrhage, the catecholamine surge occurring at the time of cerebral bleeding may cause a severe cardiac and pulmonary reversible dysfunction. Similarly, the intensive sympathetic activity and catecholamine release associated with brain death may result in severe myocardial dysfunction [5] originating from multiple factors: calcium overload [18], a possible reduction in high-energy phosphates [19], beta-adrenoreceptor desensitization, endothelial damage [19,20], and altered gene expression. Further decreased thyroid hormone (especially T3, insulin, and cortisol levels) are seen [13]. Pituitary failure produces abnormal temperature homeostasis, and eventually a catecholamine-deficient vasoparetic state occurs. All these phenomena may further affect cardiac function.

### Impact of ‘optimal donor management’

Management of ‘marginal’ hearts should include donor graft ‘resuscitation’ and re-evaluation [3,5], thus allowing potential organ rescue and utilization. Many authors and guidelines [6] support treatment with insulin, corticosteroids [21], T3 [22] and arginine vasopressin [23] which may improve ventricular performance, raise systolic blood pressure and reduce inotropic requirements, obtaining early and sufficient circulatory stabilization. Published controlled studies do not support HT treatment in stable potential donors but other prospective results are needed to investigate HT effects in hemodynamically unstable donors, considering longer duration of treatment [24]. However, in non-randomized clinical studies, organs that were at first glance assessed as marginal and/or unacceptable had the potential to improve with such an integrated approach and thus be utilized, resulting in an increase of utilization rates from only 39% to 58% [21], with excellent results in experienced centers.

### Study limitations

This is a single-center experience with a relatively low sample size. In addition, the observational, not randomized, study design does not allow us to separate the effects of HT (treat-wait-and-see) vs simple wait-and-see strategy on the observed benefit. In fact, neurogenic stunning can also spontaneously disappear with time, as clearly shown in patients with subarachnoid hemorrhage and Takotsubo cardiomyopathy [13]. We used visual assessment of left ventricular ejection fraction (LVEF) despite general recommendations to use quantitative biplane Simpsons (BPS) measurements. Although quantitative methods are well-validated and to be preferred in a research-oriented setting, visual assessment (eyeballing) is unquestionably easier and faster, and possibly even more accurate, especially in a technically demanding clinical setting such as the neurological intensive care unit with brain dead potential donors, with poor image quality, and when a well-trained observer blinded to the study condition performs the analysis [11].

### Clinical implications

This study shows that it is possible to provide long maintenance periods based on high quality intensive care management without losing the possibility of organ retrieval. Consequently, the concept of “maintenance” of potential organ donors could be extended to “treatment” of a malfunctioning graft in the potential donor before organ retrieval. Larger, multicenter, prospective studies are needed to evaluate the effects of hormonal treatment and timing on potential organ donors under conditions of hemodynamic instability. This approach can be added to other strategies proposed to expand the heart donor pool, such as the ADONHERS project, with stress echo-driven selection of old donor hearts for heart transplantation [25-28]. Using stress echo, ADONHERS recruits hearts previously excluded from transplantation due to advanced age, whereas the approach proposed here utilizes serial TTE to rescue hearts previously excluded for resting left ventricular wall motion abnormalities. At least in theory, the stress echo approach might also be applied to these potential heart donors with left ventricular abnormalities, since viability response during stress echo effectively recognizes viable tissue with non-fixed response, as opposed to necrotic response with scar and fixed wall motion abnormalities following inotropic challenge with either dobutamine or dipyridamole [29]. Information on resting function, viability and ischemia can all be obtained in a one-stop shop with pharmacologic stress echocardiography in a bedside, low-cost, and radiation-free approach [30].

Donor hearts with recovered LV function can be eligible for uneventful heart transplant. A strategy based on a) longer interval of maintenance of the brain-dead potential donor (wait), b) a circulatory targeted treatment including HT (treat) and c) heart function monitoring by 2D echo (see) can help to rescue organs suitable for heart donation.

## Abbreviations

BD, brain death; BPS, biplane Simpsons; CVP, central venous pressure; DBD, brain-dead potential donors; EF, Ejection Fraction; HT, Hormonal Treatment; LV, left ventricular; MAP, mean arterial pressure; NE, norepinephrine; PaO2, partial pressure of oxygen; PGF, Primary graft failure; T3, triiodothyronine; 2-DE, 2-dimensional echo.

## Competing interests

The authors declare that they have no competing interests.

## Authors' contributions

MC and FP conceived this study, performed the data analysis, and drafted the manuscript; TB gave a contribution to the preparation of study design, data discussion, and critical revision of the manuscript; DS and MGG were responsible for data collection and revised the manuscript. All authors read and approved the final manuscript.

## Authors' information

MC, Transplant Coordinator, Neuro Intensive Care Unit, University City Hospital, Verona, Italy.

TB, Scientific Coordinator of the CCM project n. 48 “ Aged Donor Heart Rescue by Stress Echo – ADONHERS” Institute of Clinical Physiology, National Research Council, Pisa, Italy. DS, Neuro Intensive Care Unit, University City Hospital, Verona, Italy.

MGG, Echocardiography, Cardiology, University City Hospital, Verona, Italy.

FP, Intensive Care Coordination, National Transplant Centre, Italian National Institute of Health, Roma, and Director, Neuro Intensive Care Unit, University City Hospital, Verona, Italy.

## Supplementary Material

Additional file 1 Title of data. Serial four chamber cineloops of a potential heart donor with hemodynamic instability. Description of data: Left upper panel Normal LV function before brain death. Right upper panel. Takotsubo-like LV dysfunction immediately after subarachnoid hemorrhage and brain death: echocardiographically detected left ventricular systolic dysfunction excludes this heart from transplant according to standard criteria. Left lower panel Reduction of end-systolic volume and improved systolic thickening. One day after early and aggressive hormonal treatment, including triiodothyronine, vasopressin, insulin and methylprednisolone with improvement of heart function. Right lower panel. Two days after hormonal treatment the heart definitively normalized, was retrieved and was successfully transplanted. Click here for file
